# Antibody–Drug Conjugates for the Treatment of Non-Small Cell Lung Cancer with Central Nervous System Metastases

**DOI:** 10.3390/curroncol31100471

**Published:** 2024-10-18

**Authors:** David J. H. Bian, Sara F. Cohen, Anna-Maria Lazaratos, Nathaniel Bouganim, Matthew Dankner

**Affiliations:** 1 Department of Internal Medicine, Faculty of Medicine and Health Sciences, McGill University, Montreal, QC H3A 1G1, Canada; david.bian@mail.mcgill.ca; 2 Department of Anatomy & Cell Biology, McGill University, Montreal, QC H3A 1G1, Canada; sara.cohen2@mail.mcgill.ca; 3 Faculté de Médecine, Université de Montreal. Montreal, QC H3A 1G1, Canada; anna-maria.lazaratos@mail.mcgill.ca; 4 Rosalind and Morris Goodman Cancer Institute, McGill University, Montreal, QC H3A 1G1, Canada; 5 Department of Oncology, McGill University Health Centre, Montreal, QC H3A 1G1, Canada; nathaniel.bouganim@mcgill.ca

**Keywords:** brain metastasis, central nervous system metastasis, non-small-cell lung cancer, lung cancer, antibody–drug conjugate, ADC

## Abstract

Antibody–drug conjugates (ADCs) represent an emerging class of targeted anticancer agents that have demonstrated impressive efficacy in numerous cancer types. In non-small cell lung cancer (NSCLC), ADCs have become a component of the treatment armamentarium for a subset of patients with metastatic disease. Emerging data suggest that some ADCs exhibit impressive activity even in central nervous system (CNS) metastases, a disease site that is difficult to treat and associated with poor prognosis. Herein, we describe and summarize the existing evidence surrounding ADCs in NSCLC with a focus on CNS activity.

## 1. Introduction

Lung cancer is diagnosed in approximately 32,000 Canadians each year and causes approximately 21,000 deaths annually, rendering it the number one cause of cancer-related deaths in Canadian men and women [1]. Globally, lung cancer is similarly the most common cancer with an incidence rate of over 2,400,000 cases and a mortality rate of over 1,800,000 deaths in 2022 [2]. The central nervous system (CNS) is a frequent site of metastasis in both small cell lung cancer and non-small cell lung cancer (NSCLC) and is associated with a poor prognosis [3,4,5]. CNS metastases include metastases to the brain parenchyma (denoted as brain metastases (BM)), as well as leptomeningeal metastases (LM), which involve cancer cells reaching the subarachnoid space that harbors the cerebrospinal fluid surrounding the brain and spinal cord [6].

The current management of CNS metastases is multifaceted. Current modalities for treatment include surgical resection, radiation therapy approaches, such as stereotactic radiosurgery (SRS) and whole-brain radiotherapy (WBRT), and systemic therapies including chemotherapy, targeted therapies, and immunotherapies [7,8]. Surgical approaches are generally reserved for NSCLC patients with one or few large and symptomatic BM [9].

The medical management of NSCLC BM remains challenging. The blood–brain barrier (BBB) and efflux transporters in the brain endothelium represent significant impediments for traditional chemotherapies from accessing BM lesions [10,11]. However, small molecules targeting oncogenic driver mutations in NSCLC, such as epidermal growth factor receptor (EGFR) (osimertinib, gefitinib, erlotinib, amivantanab/lazertinib), anaplastic lymphoma kinase (ALK) (crizotinib, ceritinib, brigatinib, lorlatinib), ROS1 (entrectinib, lorlatinib), hepatocyte growth factor (MET) exon 14 skipping mutations (ex14mut) (capmatinib, tepotinib), rearranged during transfection (RET) fusions (selpercatinib), v-raf murine sarcoma viral oncogene homolog B1 (BRAF) V600E (dabrafenib/trametinib), Kirsten rat sarcoma virus (KRAS) G12C (sotorasib), and neurotrophic tyrosine receptor kinase (NTRK) fusions (entrectinib, larotrectinib) have demonstrated activity in both LM and BM [12,13,14,15,16,17,18,19,20,21,22,23,24,25]. Immune checkpoint inhibitors (ICI) have similarly demonstrated efficacy in patients with NSCLC CNS metastases, particularly when combined with radiotherapy [26]. Interestingly, preclinical data suggest a mechanism of action for immunotherapy in BM whereby immune cells are primed against CNS metastases via exposure to cancer cells in the primary tumor or extracranial metastases following treatment with immunotherapy before homing to CNS metastases [27].

Antibody–drug conjugates (ADCs) represent a novel class of anticancer drugs that have arrived at the forefront of new treatment strategies for NSCLC in recent years with an emerging interest for its use in CNS metastases [28,29]. In this review, we will summarize and describe the literature evaluating ADCs in NSCLC, with a particular focus on BM.

## 2. Antibody–Drug Conjugates

ADCs are comprised of three key components: a monoclonal antibody targeting a tumor-expressed antigen, a cytotoxic payload, and a cleavable linker. These key components of an ADC play important roles in establishing its therapeutic, pharmacological, and toxicity profiles.

### 2.1. Antibodies and Antigens

The antibody moiety of an ADC molecule plays a significant role in its pharmacology, including its plasma circulation time, target specificity, and immunogenicity. The landscape of approved ADCs predominantly includes full-size immunoglobulin G (IgG), particularly IgG1 [30]. The IgG1 subclass offers a long serum half-life and robust Fc-mediated effector functions, including antibody cell- and complement-mediated cytotoxicity, and antibody-dependent cellular phagocytosis [31]. Murine and chimeric antibodies used in the design of early ADCs have been largely replaced with humanized variants to minimize immunogenic adverse effects [32].

The selection of a suitable target antigen for these antibody moieties has also proven to be critical to the successful development of effective ADC molecules. An ideal target can be described as one that is highly expressed on malignant cells and minimally expressed on healthy cells [33]. Current targets of ADCs for NSCLC that are either approved or in late-stage clinical development include human epidermal growth factor receptor 2 (HER2), trophoblast cell-surface antigen 2 (TROP2), human epidermal growth factor receptor 3 (HER3), and MET [34]. ADCs directed against these targets are currently being clinically evaluated as both monotherapy and in combination with other agents, such as immune checkpoint inhibitors (ICI) [34].

Several of these targets are significant in the context of BM given expression patterns of the target antigens specifically in CNS lesions. HER2 and HER3 have both been found to be consistently overexpressed in breast and NSCLC BM, representing targets poised for clinical activity in this setting [35,36,37,38,39,40]. MET is also commonly overexpressed or amplified in NCSLC BM, potentially establishing MET as an important functional mediator of BM that can be targeted for therapeutic benefit [41,42,43,44]. Together, the antigen targets of many of the ADCs that are approved or in the late stages of development for NSCLC are primed for promising activity in the CNS given their overexpression in BM.

### 2.2. Linkers

Linkers are molecular sequences that covalently connect the antibody to a cytotoxic payload. Their main roles are to prevent the premature release of the payload in the circulation while ensuring its release at the target site. Linkers can be broadly categorized into either cleavable or non-cleavable variants depending on the release mechanisms of their cytotoxic payloads [45]. Cleavable linkers are versatile and widely employed in ADC development. These linkers chemically release the payload through reduction, proteolysis, or hydrolysis based on specific tumor cell–associated processes, such as acidification, glutathione reduction, or lysosomal protease activity [46,47]. Non-cleavable linkers consist of chemical structures that are not fragmented by enzymatic degradation. They resist conventional biochemical processes and require complete lysosomal degradation for payload release. This increases the likelihood of payload release within the target cells while minimizing cytotoxic effects on healthy cells [48]. Non-cleavable linkers generally result in less toxicity and have a longer half-life when compared to cleavable linkers [32,49]. One major disadvantage of non-cleavable linkers, however, is the lack of antitumor activity against adjacent neoplastic cells that may not express the target antigen of the ADC (i.e., bystander killing) [50].

### 2.3. Cytotoxic Payloads

Payloads are the cytotoxic component with the direct tumor-killing activity. Ideally, the payload must have a low molecular weight, high stability, and high cytotoxicity [49,51]. In fact, payloads used in ADCs are generally 100- to 1000-fold more potent than chemotherapies because they are designed to specifically target cancer cells [49]. To achieve potent cytotoxic activity, recent ADC design strategies have focused on the optimization of the drug–antibody ratio (DAR) [33]. The DAR is the average number of payload molecules conjugated to each antibody molecule, with common DARs ranging from 2 to 8 [52]. Although ADCs with higher DARs have expectedly greater in vitro potency, preclinical studies suggest that these molecules may be subject to unfavorable pharmacokinetic properties, including increased hepatic clearance and less favorable toxicity profiles that lead to narrower therapeutic indices [53].

General categories of cytotoxic payloads include tubulin inhibitors and DNA-damaging agents. Examples of tubulin inhibitors are the auristatins (e.g., MMAE and MMAF) and the maytansinoids (e.g., DM1 and DM4). These agents disrupt the assembly and disassembly of microtubules, leading to cell cycle arrest and apoptosis [49,51,54]. DNA-damaging drugs act as crosslinkers and alkylators. The most used DNA-damaging payloads are topoisomerase I inhibitors, such as camptothecin derivatives. Non-chemotherapeutic payloads such as immunostimulatory agents that generate an antitumoral immune response and radionuclides that deliver cytotoxic radiation to tumor cells are also being developed [55,56].

## 3. Antibody–Drug Conjugates and Brain Metastases

ADCs have become a part of the standard of care for various cancer types, including breast, gynecological, urological, and hematological cancers [57]. Because patients with active and/or untreated BM are often excluded from clinical trials, the intracranial activity of ADCs specifically for CNS metastases has been largely undefined [28]. However, several recent clinical trials in breast cancer have evaluated the ADC trastuzumab deruxtecan (TDXd) specifically for patients with BM, demonstrating encouraging results. The phase II TUXEDO-1 trial evaluated TDXd in 15 HER2-positive breast cancer patients with active BM who were either previously untreated or were refractory to local treatment [58]. The trial demonstrated an intracranial overall response rate (ORR) of 73.3% and a median progression-free survival (mPFS) of 21.0 months [59,60]. The phase II DEBBRAH trial evaluated TDXd in HER2-positive breast cancer in distinct cohorts, including patients with stable BM treated with local therapy, patients with asymptomatic and untreated BM, patients with progressive BM refractory to local therapy, and patients with LM. The first data read out from this study, which excluded the LM cohort, demonstrated an intracranial ORR of 66.7% and a 6-month PFS rate of 78.7% [61]. Separate publication of the LM cohort demonstrated an mPFS of 8.9 months and a median overall survival (mOS) of 13.3 months [62]. Most recently, the DESTINY-Breast12 study enrolled 263 patients with HER2-positive breast cancer and BM. CNS-specific ORR and 12-month CNS-specific PFS were, respectively, 79.2% and 57.8% in patients with stable BM and 62.3% and 60.1% in patients with active BM [63]. Thus far, no clinical trials have been performed with ADCs in NSCLC specifically for patients with CNS metastases. However, multiple studies have included subsets of patients with BM.

## 4. HER2 Antibody–Drug Conjugates

HER2 is a transmembrane receptor tyrosine kinase encoded by the *ERBB2* gene that, under normal conditions and upon ligand binding by its dimerization partner (e.g., EGFR or HER3), heterodimerizes to activate a variety of downstream signaling pathways such as the phosphoinositide 3-kinases (PI3K) and the mitogen-activated protein kinase (MAPK) pathways. When HER2 is mutated or amplified during tumorigenesis, it becomes constitutively activated in the absence of ligand binding, leading to enhanced dimerization resulting in downstream signaling and cell proliferation [64]. In NSCLC, *HER2* is implicated as an oncogene via amplification, protein overexpression, or *HER2* mutations, the most common of which are exon 20 insertion mutations [65,66].

Approximately 2% of tumors from patients with NSCLC harbor *HER2*-activating mutations [64]. *HER2* mutations in NSCLC are associated with young age, female sex, never smoker status, and advanced, more aggressive disease that is associated with poor prognoses [67,68,69,70,71,72,73]. Patients with *HER2*-mutant NSCLC have a high incidence of BM, with point estimates between 9–32% of patients, making these patients more likely to develop BM than *KRAS* or *EGFR*-mutant NSCLC [66,67,68,69,70,71,74,75].

*HER2* is amplified in 2–5% of patients with NSCLC, and the prevalence of BM in these patients is between 2 and 22% [65,73,76,77]. In the 2–30% of patients with NSCLC whose tumor has HER2 protein overexpression, the prevalence of BM is approximately 7–23% [72,73,76]. Compared to *HER2*-mutant NSCLC, patients with NSCLC whose tumors have *HER2* amplification or protein overexpression are not associated with any specific clinicopathological features, such as age, sex, smoking status, or stage at diagnosis [66,72]. Furthermore, the prognosis of NSCLC patients with *HER2* amplification appears to be similar to that of NSCLC patients with *HER2* mutations [78].

### 4.1. Trastuzumab Emtansine

**Figure 1:**
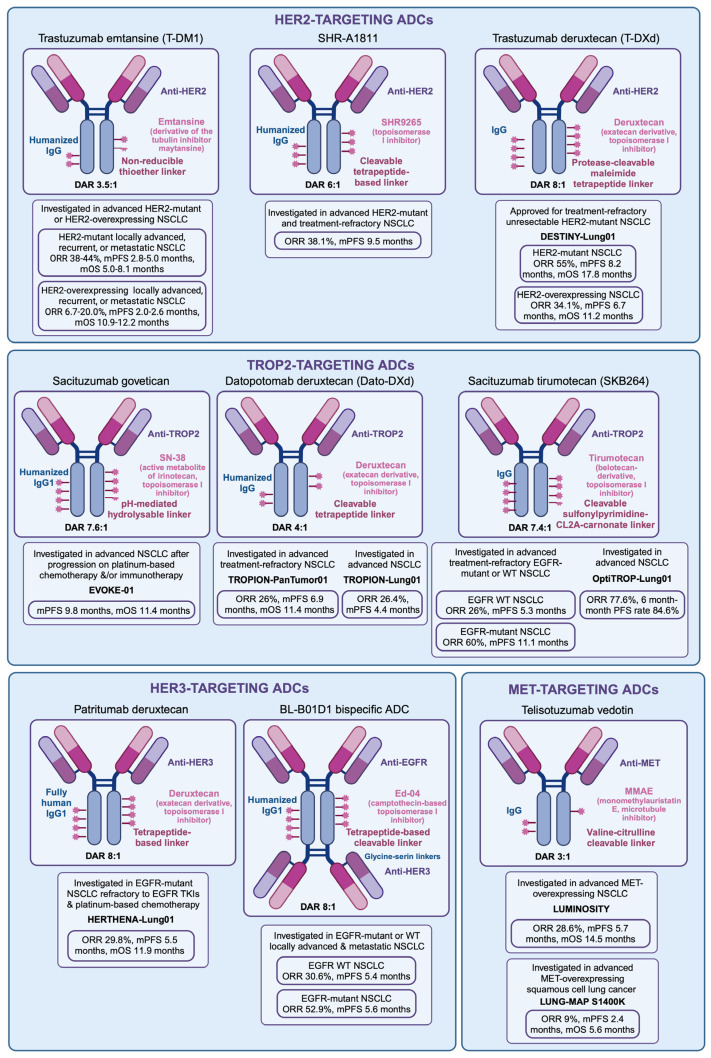
Antibody–drug conjugates evaluated for non-small cell lung cancer. HER2-targeting antibody–drug conjugates (ADCs) include trastuzumab emtansine (T-DM1), SHR-A1811, and trastuzumab deruxtecan (TDXd). TROP2-targeting ADCs include Sacituzumab govetican, datopotomab deruxtecan (Dato-DXd), and Sacituzumab tirumotecan. HER3-targeting ADCs include patritumab deruxtecan and the EGFR-HER3 bispecific ADC BL-B01D1. MET-targeting ADC includes Telisotuzumab vedotin. Outcomes for patients with non-small cell lung cancer are described. Abbreviations. ADC, antibody–drug conjugate; DAR, drug-to-antibody ratio; IgG, immunoglobulin G; mOS, median overall survival; mPFS median progression-free survival; NSCLC, non-small cell lung cancer; ORR, overall response rate. Original figure made with Biorender©.

Trastuzumab emtansine (T-DM1) is an ADC with a DAR of 3.5:1 that consists of trastuzumab, a humanized anti-HER2 IgG1 monoclonal antibody, and emtansine, a derivative of the tubulin inhibitor drug maytansine, which are linked via a nonreducible thioether linker (Figure 1) [79]. While it has been primarily studied in HER2-amplified breast cancer, it has also been investigated in patients with advanced-stage NSCLC, including those with *HER2* mutations and HER2 overexpression [80,81,82,83,84,85,86,87]. In *HER2*-mutant NSCLC patients with locally advanced, recurrent, or metastatic disease, T-DM1 showed an ORR of 38–44%, an mPFS of 2.8–5.0 months, and an mOS of 5.0–8.1 months (Figure 1) [84,85,88]. In patients with HER2 overexpressing locally advanced, recurrent, or metastatic NSCLC, T-DM1 demonstrated an ORR of 6.7–20.0%, an mPFS of 2.0–2.6 months, and an mOS of 10.9–12.2 months (Figure 1) [86,87,88]. Together, these results suggest a limited role for T-DM1 in *HER2*-altered NSCLC.

To date, no clinical trials using T-DM1 for patients with NSCLC have included subgroup analyses on patients with BM, nor have there been studies specifically analyzing this patient population. However, in patients with HER2-positive breast cancer with BM, the KAMILLA trial, which studied patients with advanced HER2-positive breast cancer, also included BM patients with untreated and asymptomatic or previously irradiated and controlled disease. The study has shown an ORR of 21.4%, an mPFS of 5.5 months, and an mOS of 18.9 months with T-DM1 [83]. Furthermore, a subgroup analysis of the DESTINY-Breast03 trial comparing T-DM1 to TDXd in patients with HER2-positive breast cancer demonstrated a clinical inferiority of T-DM1 versus TDXd [82].

### 4.2. Trastuzumab Deruxtecan

TDXd is an ADC with a DAR of 8:1 consisting of trastuzumab and deruxtecan (DXd), an exatecan with potent topoisomerase I inhibitory activity (Figure 1) [50]. The antibody and payload are linked by a protease-cleavable maleimide tetrapeptide linker, which is cleaved through lysosomal enzyme activity during ADC internalization.

TDXd has been granted accelerated approval by the U.S. Food and Drug administration (FDA) for treatment-refractory, unresectable *HER2*-mutated NSCLC, as well as for adult patients with unresectable or metastatic HER2-positive solid tumors who have received prior systemic therapy and have no alternative treatment options [89,90].

**Figure 2:**
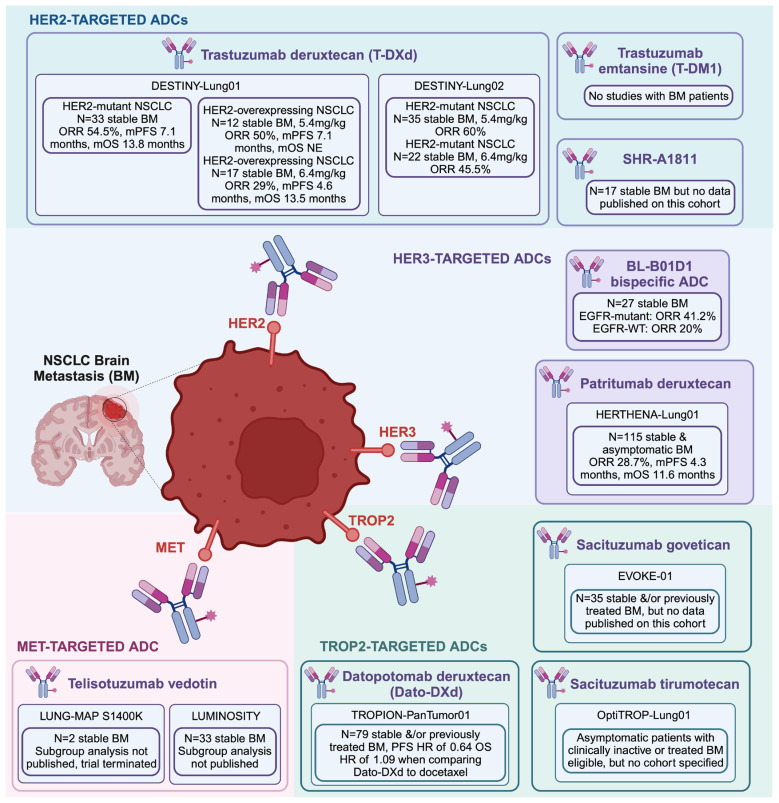
Antibody–drug conjugates evaluated for non-small cell lung cancer brain metastases.Outcomes for patients with non-small cell lung cancer brain metastases are described. Abbreviations. ADC, antibody–drug conjugate; BM, brain metastasis; HR, hazard ratio; mOS, median overall survival; mPFS median progression-free survival; NE, not estimable; NSCLC, non-small cell lung cancer; ORR, overall response rate. Original figure made with Biorender©.

The phase II DESTINY-Lung01 study investigated TDXd in patients with either *HER2*-mutant or HER2-overexpressing NSCLC. In patients whose tumors harbored *HER2* mutations, TDXd elicited an ORR of 55%, an mPFS of 8.2 months, and an mOS of 17.8 months (Figure 1) (Table 1). Importantly, the DESTINY-Lung01 study included a cohort of *HER2*-mutant NSCLC patients with stable BM, comprising 33 of the 91 (36.3%) patients included in the study (Figure 2) (Table 1). These patients experienced an ORR of 54.5%, an mPFS of 7.1 months, and an mOS of 13.8 months, results that are comparable to the cohort-at-large (Table 1) (Figure 2) [89].

The DESTINY-Lung01 trial also included a cohort of patients with treatment-refractory advanced NSCLC with HER2 overexpression. This arm of the trial included two dosing regimens with TDXd given at 5.4 mg/kg and 6.4 mg/kg. In the cohort-at-large, the 5.4 mg/kg regimen produced an ORR of 34.1%, an mPFS of 6.7 months, and an mOS of 11.2 months, while the 6.4 mg/kg regimen resulted in an ORR of 26.5%, an mPFS of 5.7 months, and an mOS of 12.4 months (Figure 1) (Table 1). Of the patients included in the study, 12 out of 41 (29.3%) in the 5.4 mg/kg cohort and 17 of 49 (34.7%) in the 6.4 mg/kg cohort had stable BM (Figure 2) (Table 1). In this subgroup of BM patients, the 5.4 mg/kg regimen showed an ORR of 50.0%, an mPFS of 7.1 months, and an mOS that was not reached, while the 6.4 mg/kg regimen showed an ORR of 29.0%, an mPFS of 4.6 months, and an mOS of 13.5 months (Figure 2) (Table 1) [91].

This study was followed by the DESTINY-Lung02 trial, which was performed to compare clinical benefits of TDXd with 5.4 mg/kg versus 6.4 mg/kg dosing in patients with advanced NSCLC and *HER2* mutations. In the cohort-at-large, the ORR was 49% and 56% in patients receiving 5.4 mg/kg and 6.4 mg/kg of TDXd, respectively (Figure 1) (Table 1). Of the patients included in the study, 35 out of 102 (34.3%) in the 5.4 mg/kg cohort and 22 of 50 (44.0%) in the 6.4 mg/kg cohort had stable BM (Figure 2) (Table 1). In this cohort of BM patients, the ORR was 60% and 45.5% in patients receiving 5.4 mg/kg and 6.4 mg/kg of TDXd, respectively (Figure 2) (Table 1) [92].

**Table 1:** Summary of available clinical trials of antibody–drug conjugates in non-small cell lung cancer patients containing survival data of brain metastases.

Trial	PMID	Patient Population	Treatment Arms	CNS Metastasis Eligibility	ALL PATIENTS						PATIENTS WITH BM					
					Population Size (N)	ORR	DCR	Median DOR	Median PFS	Median OS	Population Size (N)	ORR	DCR	Median DOR	Median PFS	Median OS
HERTHENA-Lung01	PMID: 37689979 [93]	Previously treated patients (EGFR-TKI or platinum-based chemotherapy) with locally advanced or metastatic NSCLC with *EGFR*-activating mutations (exon 19 deletion or L858R)	Patritumab deruxtecan 5.6 mg/kg	Stable BM	225	29.8%	73.8%	6.4 months	5.5 months	11.9 months	115	28.7%	70.4%	5.5 months	4.3 months	11.6 months
U31402-A-U102	PMID: 34548309 [94]	Previously treated patients (EGFR-TKI) with locally advanced or metastatic NSCLC with *EGFR*-activating mutations	Patritumab deruxtecan 5.6 mg/kg	Stable BM	57	39.0%	72.0%	6.9 months	8.2 months	NA	25	32.0%	NA	NA	NA	NA
BL-B01D1	PMID: 38823410 [95]	Locally advanced or metastatic patients with solid tumors, including NSCLC for which no standard treatment was available	BL-B01D1 dose escalation to 3.0 mg/kg, 3.5 mg/kg, or 6.0 mg/kg	Stable BM	mEGFR: 40; wt EGFR: 62	mEGFR: 52.9%; wtEGFR: 30.6%	mEGFR: 87.5%; wtEGFR: 87.1%	mEGFR: 8.5 months; wtEGFR: NE	mEGFR: 5.6 months; wtEGFR: 5.4 months	NA	mEGFR: 17; wtEGFR: 10	mEGFR: 41.2%; wtEGFR: 20.0%	mEGFR: 100.0%; wtEGFR: 100.0%	NA	NA	NA
DESTINY Lung-01	PMID: 38547891 [91]	Unresectable or metastatic NSCLC patients that was refractory to standard treatment with HER2 overexpression	Trastuzumab deruxtecan 5.4 mg/kg versus 6.4 mg/kg	Stable BM	5.4 mg/kg 41; 6.4 mg/kg: 49	5.4 mg/kg: 34.1%; 6.4 mg/kg: 26.5%	5.4 mg/kg: 78.0%; 6.4 mg/kg: 69.4%	5.4 mg/kg: 6.2 months; 6.4 mg/kg: 5.8 months	5.4 mg/kg: 6.7 months; 6.4 mg/kg: 5.7 months	5.4 mg/kg: 11.2 months; 6.4 mg/kg: 12.4 months	5.4 mg/kg 12; 6.4 mg/kg: 17	5.4 mg/kg: 50.0%; 6.4 mg/kg: 29.0%	NA	NA	5.4 mg/kg: 7.1 months; 6.4 mg/kg: 4.6 months	5.4 mg/kg: NE; 6.4 mg/kg: 13.5 months
DESTINY Lung-01	PMID: 34534430 [89]	Unresectable or metastatic NSCLC patients that was refractory to standard treatment with HER2 mutations	Trastuzumab deruxtecan 6.4 mg/kg	Stable BM	91	55.0%	92.0%	9.3 months	8.2 months	17.8 months	33	54.5%	NA	NA	7.1 months	13.8 months
DESTINY Lung-02	PMID: 37694347 [92]	Unresectable or metastatic NSCLC patients that was previously treated with standard treatment with HER2 mutations	Trastuzumab deruxtecan 5.4 mg/kg versus 6.4 mg/kg	Stable BM	5.4 mg/kg: 102, 6.4 mg/kg: 50	5.4 mg/kg: 49%, 6.4 mg/kg: 56%	5.4 mg/kg: 93.1%, 6.4 mg/kg: 92.0%	5.4 mg/kg: 16.8 months, 6.4 mg/kg: NE	5.4 mg/kg: 9.9 months, 6.4 mg/kg: 15.4 months	5.4 mg/kg: 19.5 months, 6.4 mg/kg: NE	5.4 mg/kg: 35, 6.4 mg/kg: 22	5.4 mg/kg: 60%, 6.4 mg/kg: 45.5%	NA	NA	NA	NA

Abbreviations. BM, brain metastases; CNS, central nervous system; DCR, disease control rate; DOR, duration of response; HER2, human epidermal growth factor receptor 2; kg, kilogram; mg, milligram; mEGFR, mutant epidermal growth factor receptor; NA, not available; NE, not estimable; NSCLC, non-small cell lung cancer; ORR, overall response rate; OS, overall survival; PFS, progression-free survival; PMID, PubMed identifier; TKI, tyrosine-kinase inhibitor; wtEGFR, wild-type EGFR.

Importantly, CNS-specific outcomes were not published for the DESTINY-Lung01, nor the DESTINY-Lung02 trials. Despite this, the findings from the DESTINY-Lung01 and DESTINY-Lung02 studies suggest that patients with stable BM experience systemic ORR and PFS that is similar to the cohort-at-large, and that the additional toxicity seen with the 6.4 mg/kg dose does not add additional therapeutic activity beyond what was observed with 5.4 mg/kg in both *HER2*-mutant and overexpressing NSCLC.

Outside of NSCLC, TDXd has demonstrated impressive efficacy in the CNS when studied in prospective and retrospective cohorts of breast cancer patients who had stable or active and progressive CNS metastases [58,60,61,62,63,96,97]. These findings, in combination with the DESTINY-Lung01 and DESTINY-Lung02 trials, suggest that patients with *HER2*-mutated or overexpressing BM may be uniquely poised to experience similar treatment outcomes compared to patients without BM when treated with TDXd. While DESTINY-Lung01 and DESTINY-Lung02 are single-armed studies, it is apparent that *HER2*-mutated NSCLC patients would likely experience superior outcomes when treated with TDXd compared to an alternative standard of care [89,91,92,98]. Importantly, the same cannot be said for patients with NSCLC whose extracranial tumors overexpress HER2, for whom ORR and mPFS in the DESTINY-Lung01 trial are closer to historical controls [91,98]. For this reason, the subgroup of patients with HER2-overexpressing NSCLC who specifically have CNS metastases, after further study, may be uniquely positioned to derive benefit from TDXd compared to alternative agents that possess poor intracranial activity

### 4.3. Other HER2-Targeting ADCs

Novel ADCs targeting HER2 alterations in NSCLC are currently being investigated in clinical trials. SHR-A1811 is a novel HER2-targeting ADC consisting of a trastuzumab antibody conjugated with a novel DNA topoisomerase I inhibitor payload (SHR9265) by a cleavable tetrapeptide-based linker (Figure 1). With a DAR of 6:1, SHR-A1811 has demonstrated superior membrane permeability, cytotoxicity, and antitumor activity in in vitro and in vivo preclinical models when compared with TDXd. Results from a phase I/II trial investigating SHR-A1811 in *HER2*-mutated advanced-staged and treatment-refractory NSCLC patients have shown an ORR of 38.1% and an mPFS of 9.5 months (Figure 1). Of the patients included in the study, 17 out of 63 (27.0%) had stable BM at enrollment, but no subgroup analysis on the survival outcomes of these patients has been published to date [99].

## 5. HER3 Antibody–Drug Conjugates

HER3, encoded by the *ERBB3* gene, is a receptor tyrosine pseudokinase that has no intrinsic kinase activity but binds to ligands and dimerizes with other receptor tyrosine kinases, such as the ligand binding-impaired HER2 [100,101]. The overexpression of HER3 has been identified in a number of solid tumors in both pre- and post-treatment settings, including NSCLC [102]. HER3 expression can be identified by immunohistochemistry in most NSCLC specimens, and HER3 has consistently been found to be overexpressed in BM when compared to matched and unmatched primary tumors [36,38,39,103,104].

### 5.1. Patritumab Deruxtecan

Patritumab deruxtecan (HER3-DXd) is an ADC comprised of patritumab, a humanized anti-HER3 IgG1 monoclonal antibody, and deruxtecan. The two components are covalently linked with a tetrapeptide-based linker, with HER3-DXd having a DAR of 8:1 (Figure 1) [105].

HER3-DXd has been studied in clinical trials that included patients with breast cancer and NSCLC [93,94,106,107]. In NSCLC, the phase II HERTHENA-Lung01 study included patients with EGFR mutations who were refractory to EGFR–tyrosine kinase inhibitors (TKIs) and platinum-based chemotherapy. The trial demonstrated an ORR of 29.8%, an mPFS of 5.5 months, and an mOS of 11.9 months (Figure 1) (Table 1). Of the patients included in the trial, 115 out of 225 (51.1%) had stable and asymptomatic BM, with an ORR of 28.7%, a median duration of response (mDOR) of 5.5 months, an mPFS of 4.3 months, and an mOS of 11.6 months in this subgroup, which is comparable to the cohort-at-large (Figure 2) (Table 1). Of note, 30 included patients had BM on diagnosis of NSCLC that was not pretreated with intracranial radiotherapy. This subgroup demonstrated an ORR of 33.3%, and an mDOR of 8.4 months [93]. The phase III trial HERTHENA-Lung02 is currently underway, evaluating patients with advanced NSCLC refractory to third-generation EGFR-TKIs, and also permits patients with stable BM to participate in the study (Table 2) [108]. The ongoing TUXEDO-03 trial is specifically tailored to evaluate HER3-DXd in patients with breast cancer or NSCLC with active BM who have received at least one previous line of systemic therapy in the advanced setting, and in metastatic solid tumor patients who have either treatment-naïve LM or post-radiation recurrent LM who do not require immediate local treatment (Table 2) [59].

### 5.2. Other HER3-Targeting Antibody–Drug Conjugates

BL-B01D1 is a first-in-class EGFR-HER3 bispecific ADC with a DAR of 8:1. BL-B01D1 consists of a bispecific humanized IgG1 anti-EGFR antibody fused to two anti-HER3 humanized single-chain fragment variables via glycine-serin linkers, a tetrapeptide-based cleavable link, and Ed-04, a camptothecin-based topoisomerase I inhibitor (Figure 1). Of note, the antibody component of BL-B01D1 can target both mutant and wild-type EGFR. A phase I study evaluated BL-B01D1 in locally advanced and metastatic NSCLC patients both with and without *EGFR* mutations, among multiple other solid tumors. The study included 102 patients with NSCLC, of which 27 (26.5%) had stable BM (Figure 2) (Table 1). In the NSCLC cohort, the ORR was 52.5% (95% confidence interval (CI): 37.5–67.1%) in patients with *EGFR* mutations and 30.6% (95% CI: 20.6–43.0%) in patients with wild-type *EGFR* (Figure 1) (Table 1). The mPFS was 5.6 months in patients with *EGFR* mutations and 5.4 months in patients with wild-type *EGFR* (Figure 1) (Table 1). In the cohort of patients with BM at the time of enrollment, the ORR was 41.2% (95% CI: 18.4–67.1%) in patients whose tumors harbored *EGFR* mutations, and 20.0% (95% CI: 2.5–55.6%) in patients with wild-type *EGFR* (Figure 1) (Table 1) [95]. A phase II trial to further characterize BL-B01D1 in metastatic NSCLC is currently underway (Table 2).

## 6. TROP2 Antibody–Drug Conjugates

TROP2 is encoded by the tumor-associated calcium signal transducer 2 (TACSTD2) gene expressed in many normal tissues [109]. TROP2 overexpression has been implicated in multiple cancers, including breast cancer, glioblastoma, and NSCLC [110].

In NSCLC, multiple studies have described TROP2 overexpression in patients with NSCLC, ranging from 42% to 100% of patients tested demonstrating intratumoral TROP2 expression by IHC [111,112,113,114]. Multiple studies have similarly shown that TROP2 overexpression has been associated with advanced tumor staging and shorter OS, particularly in lung adenocarcinomas [111,113,114,115,116]. TROP2 overexpression has also been implicated in treatment resistance and poor outcomes in NSCLC patients previously treated with ICIs [116].

In NSCLC BM, one study observed high levels of TROP2 expression at the RNA level that are ubiquitously present in resected BM of NSCLC patients, but no matched nor unmatched primary tumors were described as controls [117]. More studies are required to better understand if TROP2 overexpression status is enriched in NSCLC with BM.

### 6.1. Sacituzumab Govitecan

Sacituzumab govitecan (SG) is an ADC with a DAR of 7.6:1 consisting of a humanized anti-TROP2 IgG1 kappa antibody and the topoisomerase I inhibitor SN-38, the active metabolite of irinotecan, which are linked by a pH-mediated hydrolysable linker (Figure 1) [118]. SG has demonstrated prolonged PFS and OS compared to single-agent physicians’ choice chemotherapy in patients with metastatic triple-negative breast cancer [119,120]. In NSCLC, the phase III EVOKE-01 trial compared SG to docetaxel in patients with advanced NSCLC after progression on platinum-based chemotherapy and/or immunotherapy. In the study, patients with stable and/or previously treated BM were included with 35 out of 299 (11.7%) patients in the SG arm compared to 39 out of 304 (12.8%) patients in the docetaxel arm (Figure 1). The mOS and mPFS were not significantly higher in the SG arm versus the docetaxel arm, at 11.1 and 4.1 months versus 9.8 and 3.9 months, respectively (Figure 1). Despite the significant number of NSCLC BM patients included in the study, the authors did not describe survival outcomes in this subgroup [121].

While no studies have directly evaluated SG in NSCLC BM patients, a phase 0 clinical trial evaluated SG in 25 recurrent glioblastoma and triple negative breast cancer BM patients with treatment-refractory disease. This early phase study observed an intracranial ORR of 50% in breast cancer BM and 28% in glioblastoma, an mPFS of 8 months in breast cancer BM and 2 months in glioblastoma, and an mOS of 35.2 months in breast cancer BM and 9.5 months in glioblastoma [122].

In the context of negative survival outcomes with SG in NSCLC compared to docetaxel in the population at-large in the EVOKE-01 trial, and a lack of described subgroup analyses for the patients with BM included in the trial, it is unlikely that SG represents a viable therapeutic option with enhanced intracranial efficacy.

### 6.2. Datopotamab Deruxtecan

Datopotamab deruxtecan (Dato-DXd) is an ADC with a DAR of 4:1 consisting of the humanized anti-TROP2 IgG1 monoclonal antibody datopotamab and deruxtecan, which are linked by a cleavable tetrapeptide-based linker (Figure 1) [123]. The phase I TROPION-PanTumor01 trial evaluated Dato-DXd in advanced-stage, treatment-refractory NSCLC patients. The study demonstrated an mPFS of 6.9 months, an mOS of 11.4 months, and an ORR of 26%. Of the patients included in the study, 68 out of 180 (37.8%) had stable and previously treated BM (Figure 1). However, the authors did not describe the survival outcomes in the BM subgroup [124].

Additionally, the phase III TROPION-Lung01 study compared Dato-DXd to docetaxel in advanced-stage NSCLC patients. When comparing Dato-DXd to docetaxel, the study demonstrated, respectively, an ORR of 26.4% versus 12.8%, an mPFS of 4.4 months versus 3.7 months, and an mOS of 12.9 months versus 11.8 months (Figure 1). Furthermore, a significant portion of the included patients had previously treated or inactive BM, with 79 out of 299 (26.4%) patients in the Dato-DXd group and 91 out of 305 (29.8%) patients in the docetaxel group. When comparing the PFS of Dato-DXd to docetaxel, patients with BM had a hazard ratio (HR) of 0.64 (95% CI: 0.38–1.05) while patients without BM had a HR of 0.76 (95% CI: 0.60–0.94), both favoring Dato-DXd (Figure 2). However, when comparing the OS of Dato-DXd to docetaxel, patients with BM had a HR of 1.09 (95% CI: 0.68–1.75) and patients without BM had a HR of 0.89 (95% CI: 0.74–1.12) (Figure 2) [125]. Both Dato-DXd alone and in combination with other therapies are currently being further investigated in multiple other clinical trials (Table 2) [126].

### 6.3. Sacituzumab Tirumotecan

Sacituzumab tirumotecan (SKB264) is an ADC with a DAR of 7.4:1 consisting of the previously described sacituzumab antibody and the payload tirumotecan, a belotecan-derivative topoisomerase I inhibitor, which are bound by a cleavable sulfonyl pyrimidine-CL2A-carbonate linker (Figure 1). In in vivo preclinical studies, SKB264 demonstrated a significantly longer half-life and superior intratumor payload concentration compared to SG [127]. In patients with advanced-stage, treatment-refractory NSCLC, SKB264 demonstrated an ORR of 26.0% and an mPFS of 5.3 months in the wild-type *EGFR* NSCLC group and an ORR of 60.0%, and an mPFS of 11.1 months in the TKI-resistant *EGFR*-mutant NSCLC group (Figure 1) [128]. In the OptiTROP-Lung01 study, SKB264 was combined with KL-A167, an anti-PD-L1 agent, for the treatment of advanced-stage NSCLC as first-line therapy. This trial showed an ORR of 77.6% and a 6-month mPFS rate of 84.6% (Figure 1) [129]. According to the trial protocol, asymptomatic patients with clinically inactive or previously treated BM were eligible for the study, but subset analyses of these patients have yet to be published (Table 2) [128,129].

## 7. MET Antibody–Drug Conjugates

MET is a well-described proto-oncogene receptor tyrosine kinase [130]. MET plays a significant role in tumor proliferation, angiogenesis, invasion, and cell survival [131]. In breast cancer, MET has been implicated in BM biology through the tumor microenvironment [42,132]. In NSCLC, multiple mechanisms of MET dysregulation have been described, including ex14mut, amplification, and overexpression.

Amplifications of *MET* have been described as an adaptive resistance mechanism in *EGFR*-mutated NSCLC treated with EGFR-TKIs [102,133]. *MET* amplification in NSCLC has been associated with early development of metastasis, including BM [134]. In a recent study evaluating the genomics of NSCLC BM, the authors observed a significantly increased *MET* amplification frequency of 4.4% in NSCLC BM compared to 2.3% in unmatched NSCLC primary tumors [135]. In a study by Preusser et al. (2014), the investigators observed a *MET* amplification frequency of up to 21.6% in resected NSCLC BM samples, but no matched nor unmatched primary tumor controls were analyzed [41].

*MET* ex14mut represents a targetable oncogenic mutation with approved therapeutics in NSCLC, such as capmatinib. A recent meta-analysis demonstrated a median incidence of 15.0% of *MET* ex14mut in NSCLC patients that have BM at the time of diagnosis [136]. However, a retrospective study found a significantly lower frequency of *MET* ex14mut in resected NSCLC BMs when compared to unmatched resected NSCLC primary tumors (1.0% versus 2.3%, respectively) [135].

In NSCLC, MET protein overexpression has been estimated to be 13.7%–25.0% [137,138,139]. In NSCLC BM, the frequency of MET protein overexpression has been identified in 44.4% of resected specimens [41].

Given the high expression levels of MET in NSCLC, including in BM, whether via amplification, oncogenic mutation, or protein overexpression, MET represents a promising therapeutic target for ADC development.

### Telisotuzumab Vedotin

Telisotuzumab vedotin (Teliso-V) is an ADC with a DAR of 3:1 comprising of ABT-700, a humanized monoclonal anti-MET antibody, conjugated to monomethylauristatin E, a cytotoxic microtubule inhibitor, through a valine-citrulline linker that is cleavable by intracellular proteolysis (Figure 1). Teliso-V is notable for its in vitro antitumor activity against MET-overexpressing tumor cells with or without *MET* amplification [140]. The phase II LUMINOSITY trial evaluated Teliso-V in advanced-stage NSCLC patients with MET overexpression. Of the NSCLC patients included in the study, 33 out of 161 (20.5%) had previously treated and stable BM. The trial demonstrated an ORR of 28.6%, an mPFS of 5.7 months, and an mOS of 14.5 months in overall NSCLC patients (Figure 1). No subgroup analysis was presented for NSCLC BM patients, however [141].

The phase II LUNG-MAP S1400K trial was conducted to evaluate the efficacy of Teliso-V in advanced-stage squamous cell lung cancer patients with MET overexpression. Of the patients included in the trial, 2 out 23 (8.7%) had stable BM. The trial was terminated with an ORR of 9.0%, an mPFS of 2.4 months, and an mOS of 5.6 months [142].

The TeliMET NSCLC-1 phase III trial, which will compare Teliso-V monotherapy versus docetaxel in advanced-stage non-squamous NSCLC patients with MET overexpression, is currently underway (Table 2). Similar to the LUMINOSITY and LUNG-MAP S1440K trials, the TeliMET NSCLC-1 trial will include NSCLC patients with stable BMs.

## 8. Conclusions and Future Directions

In recent years, ADCs have become an important component of the treatment armamentarium for NSCLC, particularly in patients with HER2-mutated tumors with TDXd [89,91,92]. Meanwhile, other ADCs in development, consisting of a variety of novel molecular targets, linkers, and payloads, may soon become part of the standard of care upon further investigation. CNS metastases represent an important bottleneck limiting the improvement of survival outcomes in patients with metastatic NSCLC, creating impetus for the development of agents for patients specifically with CNS disease [143]. Third-generation EGFR inhibitors represent a class of therapeutics developed with CNS activity in mind, demonstrating impressive activity in both BM and LM [16,144,145,146]. However, EGFR mutations are only present in approximately 30% of patients with NSCLC, leaving the majority of NSCLC BM patients without therapies specifically designed for their disease site [147].

CNS metastases are unique due to a lack of activity of conventional chemotherapies in this setting. The reasons for this are believed to be multifaceted, with the BBB playing an important role in limiting drug penetration and rapidly removing a drug that enters the CNS via efflux transporters [11]. Despite the fact that, dogmatically, small molecules such as TKIs more readily penetrate CNS lesions than bulkier molecules such as antibodies, ADCs have elicited robust activity in proof-of-concept studies, such as in the TUXEDO-1 and DESTINY-Breast12 trials with TDXd in breast cancer BM [58,63].

There are several speculative hypotheses to explain why TDXd seemingly demonstrates similar outcomes in intracranial versus extracranial lesions in these studies, which may be translated to NSCLC, with future study. It is possible that, with the long circulating half-life of TDXd, DXd is slowly cleaved from the antibody, establishing a slowly released reservoir of drug able to cross the BBB and penetrate CNS lesions. It is also possible that the increased presence of HER2 dimer partners, such as HER3, or ligands in the brain microenvironment, such as neuregulin, may lead to increased HER2 internalization, favoring TDXd activity in the CNS despite it likely being present at lower concentrations compared to the systemic circulation [88]. The ongoing ELPIS study evaluating untreated, and asymptomatic BM in advanced NSCLC and breast cancer will further describe the activity of TDXd for solid tumor BM, but there is an impetus for additional studies that include patients with active BM and LM (Table 2). The lack of CNS-specific studies will continue to make strong claims about the activity of any ADC in the CNS of NSCLC patients challenging.

Beyond TDXd, it remains to be seen whether other ADCs will similarly demonstrate robust activity in CNS lesions. HER3-DXd represents a target that may similarly lead to augmented intracranial activity because of the well-established upregulation of HER3 in BM. The ongoing TUXEDO-3 study of HER3-DXd in NSCLC BM and LM will shed light as to whether this hypothesis holds true (Table 2).

There is a rapidly expanding number of ADCs that are currently under investigation for NSCLC. A list of currently ongoing prospective studies is listed in Table 2. Most of these studies exclude patients with progressive and untreated BMs, with some including stable BMs in their eligibility criteria.

Together, ADCs represent an expanding component of the therapeutic toolkit used to treat NSCLC. With further development, there is hope that these agents can be optimized to the benefit of patients with CNS metastases, who are, given their guarded prognoses, most in need of new and effective therapeutic strategies.

## Figures and Tables

**Table 2:** Summary of ongoing clinical trials evaluating antibody–drug conjugates in overall non-small cell lung cancer patients and central-nervous system–specific non-small cell lung cancer patients.

	Trial	Trial Type	National Clinical Trial Number (NCT)	Patient Population	Antibody–Drug Conjugate	Antibody Target	Payload Type	CNS Metastasis Eligibility	Status
**CNS-specific NSCLC studies**	Efficacy and Safety of T-DXd in HER2-mutant Advanced Lung Cancer Patients with Asymptomatic Brain Metastases (ELPIS)	Phase II	NCT06250777	Locally advanced and unresectable NSCLC with activating HER2 mutation and untreated asymptomatic BM at baseline	Trastuzumab deruxtecan	HER2	Topoisomerase I inhibitor	CNS metastasis-specific study	Not yet recruiting
	HER3-DXd in Breast Cancer and NSCLC Brain Metastases and Solid Tumor Leptomeningeal Disease (TUXEDO-3)	Phase II	NCT05865990	Previously treated metastatic NSCLC with newly diagnosed or progressive BM	Patritumab deruxtecan	HER3	Topoisomerase I inhibitor	CNS metastasis-specific study	Recruiting
**Non-CNS-specific NSCLC studies**	CAB-AXL-ADC Safety and Efficacy Study in Adults with NSCLC	Phase II	NCT04681131	Metastatic NSCLC	CAB-AXL-ADC	AXL	Auristatin microtubule inhibitor	Uncontrolled CNS disease excluded	Recruiting
	A Study of MORAb-202 in Participants with Previously Treated Metastatic Non-Small Cell Lung Cancer (NSCLC) Adenocarcinoma (AC)	Phase II	NCT05577715	Metastatic NSCLC with treatment-refractory progressive disease while on PD-1/L1-, EGFR-, or ALK- targeting therapies	Mecbotamab vedotin, no dosing available; mecbotamab vedotin and a PD-1 inhibitor	AXL	Auristatin microtubule inhibitor	No mention of CNS disease in the eligibility criteria	Recruiting
	A Study of BL-B01D1 and BL-B01D1 in Combination with Osimertinib Mesylate Tablets in Patients with Locally Advanced or Metastatic Non-small Cell Lung Cancer	Phase II	NCT05880706	Locally advanced, unresectable, or metastatic NSCLC	BL-B01D1	EGFRxHER3	Topoisomerase I inhibitor	Active CNS disease excluded	Recruiting
	Phase 2 Study to Investigate Luveltamab Tazevibulin in Adults with Advanced or Metastatic Non-small Cell Lung Cancer	Phase II	NCT06555263	Locally advanced, unresectable, or metastatic NSCLC with positive FOLR1 expression and treatment-refractory disease	Luveltamab tazevibulin	FOLR1	Tubulin inhibitor	Previously treated BM included; untreated CNS disease excluded	Recruiting
	Study to Investigate Luveltamab Tazevibulin in Adults with Advanced or Metastatic Non-small Cell Lung Cancer	Phase II	NCT06555263	Locally advanced, unresectable or metastatic NSCLC that is refractory to systemic therapy with positive FOLR1 expression	Luveltamab tazevibulin	FOLR1	Tubulin inhibitor	Untreated CNS disease excluded	Recruiting
	Phase Ib Study of the Safety of T-DXd and Immunotherapy Agents with and Without Chemotherapy in Advanced or Metastatic HER2+, Non-squamous NSCLC (DL03)	Phase I	NCT04686305	Locally advanced or metastatic non-squamous NSCLC that are refractory to systemic therapy or treatment-naïve with HER2 overexpression	Trastuzumab deruxtecan	HER2	Topoisomerase I inhibitor	Untreated an symptomatic CNS disease excluded	Recruiting
	A Clinical Trial of TQB2102 for Injection in Non-small Cell Lung Cancer with HER2 Gene Abnormality	Phase II	NCT06496490	Locally advanced, unresectable or metastatic NSCLC that is refractory to standard of care therapy	TQB2102	HER2	Topoisomerase I inhibitor	Stable BM included; symptomatic and progressive CNS disease, or LMD excluded	Recruiting
	HER3-DXd in Metastatic or Unresectable Non-Small Cell Lung Cancer	Phase I	NCT03260491	Locally advanced, unresectable or metastatic NSCLC with EGFR-activating mutations with progression on previously responsive EGFR-TKI treatment	Patritumab deruxtecan	HER3	Topoisomerase I inhibitor	Clinically inactive or treated and asymptomatic BM included; untreated and symptomatic CNS disease excluded	Recruiting
	HERTHENA-Lung02: A Study of Patritumab Deruxtecan Versus Platinum-based Chemotherapy in Metastatic or Locally Advanced EGFRm NSCLC After Failure of EGFR TKI Therapy	Phase III	NCT05338970	Locally advanced, or metastatic nonsquamous NSCLC with EGFR TKI treatment-refractory disease and *EGFR* mutations	Patritumab deruxtecan	HER3	Topoisomerase I inhibitor	Untreated and symptomatic BM, and history or presence of LMD excluded	Active, not recruiting
	A Study of SGN-B6A Versus Docetaxel in Previously Treated Non-small Cell Lung Cancer	Phase III	NCT06012435	Locally advanced, unresectable, or metastatic NSCLC with non-squamous histology and with progression on previous chemotherapy or targeted therapy	Sigvotatug vedotin	IB6	Auristatin microtubule inhibitor	Stable and treated BM included; active CNS disease and LMD excluded	Recruiting
	Clinical Study of Antibody-Drug Conjugate MYTX-011 in Subjects with Non-Small Cell Lung Cancer	Phase I	NCT05652868	Locally advanced, recurrent, or metastatic NSCLC with MET alterations that is refractory to the standard of care therapy	MYTX-011	MET	Auristatin microtubule inhibitor	Uncontrolled and untreated CNS disease excluded	Recruiting
	A Study to Assess Disease Activity and Adverse Events of Intravenous (IV) Telisotuzumab Vedotin Compared to IV Docetaxel in Adult Participants with Previously Treated Non-Squamous Non-Small Cell Lung Cancer (NSCLC)	Phase III	NCT04928846	Locally advanced, unresectable, or metastatic nonsquamous NSCLC with treatment-refractory disease and MET overexpression	Telisotuzumab Vedotin	MET	Microtubule inhibitor	Stable CNS disease included; new and untreated CNS disease, and LMD excluded	Recruiting
	Study of REGN5093-M114 (METxMET Antibody-Drug Conjugate) in Adult Patients with Mesenchymal Epithelial Transition Factor (MET) Overexpressing Advanced Cancer	Phase I/II	NCT04982224	Locally advanced, unresectable or metastatic NSCLC with MET overexpression	REGN5093-M114	METxMET	Microtubule inhibitor	Untreated and active CNS disease and LMD excluded	Recruiting
	First-in-Human Study of XMT-1536 in Cancers Likely to Express NaPi2b	Phase I/II	NCT03319628	Metastatic NSCLC adenocarcinoma	Upifitamab rilsodotin	NaPi2b	Auristatin microtubule inhibitor	Untreated CNS disease, such as new and progressive BM, and history of LMD excluded	Active, not recruiting
	An Efficacy and Safety Study of Cofetuzumab Pelidotin in Participants with PTK7-Expressing, Recurrent Non-Small Cell Lung Cancer	Phase I	NCT04189614	Recurrent and treatment-refractory NSCLC with PTK7 expression	Cofetuzumab pelidotin	PTK7	Auristatin microtubule inhibitor	Asymptomatic and treated CNS disease included; active CNS disease excluded	Active, not recruiting
	Datopotamab Deruxtecan (Dato-DXd) in Combination with Pembrolizumab with or Without Platinum Chemotherapy in Subjects with Advanced or Metastatic Non-Small Cell Lung Cancer (TROPION-Lung02)	Phase I	NCT04526691	Locally advanced, unresectable, or metastatic NSCLC without actionable genomic alterations (e.g., ROS1, MET, EGFR, ALK, etc.) and with treatment-refractory progressive disase	Datopotamab deruxtecan	TROP2	Topoisomerase I inhibitor	Active and untreated CNS disease excluded	Active, not recruiting
	Phase 1b Study of Dato-DXd in Combination with Immunotherapy with or Without Carboplatin in Advanced or Metastatic Non-Small Cell Lung Cancer (TROPION-Lung04)	Phase I	NCT04612751	Locally advanced, unresectable, or metastatic NSCLC without EGFR- nor ALK-mutations	Datopotamab deruxtecan	TROP2	Topoisomerase I inhibitor	Clinically active CNS disease excluded	Recruiting
	A Study of Dato-DXd in Chinese Patients with Advanced Non-Small Cell Lung Cancer, Triple-negative Breast Cancer and Other Solid Tumors (TROPION-PanTumor02)	Phase I/II	NCT05460273	Locally advanced, unresectable, or metastatic NSCLC with treatment-refractory progressive disease	Datopotamab deruxtecan	TROP2	Topoisomerase I inhibitor	LMD excluded	Active, not recruiting
	SKB264 Combination Therapy in Patients with Advanced or Metastatic Non-small Cell Lung Cancer.	Phase II	NCT05351788	Locally advanced, unresectable, or metastatic NSCLC	SKB264	TROP2	Topoisomerase I inhibitor	Active CNS metastasis, LMD, and metastases to the brainstem and spinal cord excluded	Recruiting
	Study of DS-1062a in Advanced or Metastatic Non-small Cell Lung Cancer with Actionable Genomic Alterations (TROPION-Lung05)	Phase II	NCT04484142	Locally advanced, unresectable, or metastatic NSCLC with actionable genomic alterations (e.g., ROS1, MET, EGFR, ALK, etc.) and with treatment-refractory progressive disase	Datopotamab deruxtecan	TROP2	Topoisomerase I inhibitor	Inactive BM included; untreated and symptomatic CNS disease, and LMD excluded	Active, not recruiting with results (no survival data available)
	Study of Dato-DXd Plus Pembrolizumab vs. Pembrolizumab Alone in the First-line Treatment of Subjects with Advanced or Metastatic NSCLC Without Actionable Genomic Alterations (TROPION-Lung08)	Phase III	NCT05215340	Locally advanced, unresectable, or metastatic NSCLC without actionable genomic alterations (e.g., ROS1, MET, EGFR, ALK, etc.) and with PD-L1 expression of 50% or more	Datopotamab deruxtecan	TROP2	Topoisomerase I inhibitor	Previously treated and stable BM included; active and untreated CNS disease, and LMD excluded	Recruiting
	Phase III, Open-label, First-line Study of Dato-DXd in Combination with Durvalumab and Carboplatin for Advanced NSCLC Without Actionable Genomic Alterations (AVANZAR)	Phase III	NCT05687266	Locally advanced, unresectable, or metastatic NSCLC that is not amenable to chemoradiation and without actionable genomic alterations (e.g., ALK, ROS1, MET, etc.)	Datopotamab deruxtecan	TROP2	Topoisomerase I inhibitor	Active BM and history of LMD excluded	Recruiting
	Sacituzumab Tirumotecan (MK-2870) Versus Chemotherapy in Previously Treated Advanced or Metastatic Nonsquamous Non-small Cell Lung Cancer (NSCLC) with EGFR Mutations or Other Genomic Alterations (MK-2870-004)	Phase III	NCT06074588	Locally advanced, unresectable, or metastatic non-squamous NSCLC with treatment-refractory progressive disease	Sacituzumab tirumotecan	TROP2	Topoisomerase I inhibitor	Previously treated BM included; active CNS disease and LMD excluded	Recruiting
	Study of Pembrolizumab (MK-3475) Monotherapy Versus Sacituzumab Govitecan in Combination with Pembrolizumab for Participants with Metastatic Non-small Cell Lung Cancer (NSCLC) with Programmed Cell Death Ligand 1 (PD-L1) Tumor Proportion Score (TPS) ≥50% (MK-3475-D46)	Phase III	NCT05609968	Metastatic NSCLC without an indication of EGFR-, ALK-1, or ROS-1 targeted therapies and with a PD-L1 tumor proportion score of 50% or more	Sacituzumab govitecan	TROP2	Topoisomerase I inhibitor	Active CNS disease and LMD excluded	Recruiting
**Solid tumor studies with NSCLC cohort**	A Study to Assess the Safety, Pharmacokinetics, and Antitumor Activity of BC3195 in Patients with Advanced or Metastatic Cancer	Phase I	NCT06548672	Locally advanced or metastatic solid tumors, including NSCLC, that is refractory or not amenable to the standard of care therapy	BC3195	CDH3	Auristatin microtubule inhibitor	Previously treated and stable BM included; active CNS disease and LMD excluded	Recruiting
	A Study of PF-08046050 (SGN-CEACAM5C) in Adults with Advanced Solid Tumors	Phase I	NCT06131840	Locally advanced, unresectable or metastatic solid tumors, including NSCLC, that is refractory or not amenable to the standard of care therapy	PF-08046050	CEACAM5	Topoisomerase I inhibitor	Stable and treated BM included	Recruiting
	A Phase 1 Study of CPO301 in Adult Patients with Advanced or Metastatic Solid Tumors	Phase I	NCT05948865	Advanced or metastatic solid tumors, including NSCLC, that is refractory or not amenable to the standard of care therapy	CPO301	EGFR	NA	Known, active, or uncontrolled CNS disease and LMD excluded	Recruiting
	First in Human Study of AZD9592 in Solid Tumors (EGRET)	Phase I	NCT05647122	Locally advanced or metastatic solid tumors, including metastatic NSCLC in subgroups of *EGFR*-mutant or *EGFR* wild-type disease, that is refractory or not amenable to the standard of care therapy	AZD9592	EGFRxMET	Topoisomerase I inhibitor	Stable and treated BM included; active and untreated BM and history of LMD excluded	Recruiting
	AMT-151 in Patients with Selected Advanced Solid Tumours	Phase I	NCT05498597	Advanced solid tumors, including NSCLC, that is refractory or not amenable to the standard of care therapy	AMT-151	FOLR1	NA	Untreated CNS disease excluded	Recruiting
	A Study of LY4170156 in Participants with Selected Advanced Solid Tumors	Phase I	NCT06400472	Advanced solid tumors, including NSCLC, that is refractory or not amenable to the standard of care therapy	LY4170156	FOLR1	Topoisomerase I inhibitor	Active and untreated BM, and history of LMD excluded	Recruiting
	A Study to Evaluate the Safety, Tolerability, and Efficacy of MORAb-202 (Herein Referred to as Farletuzumab Ecteribulin), a Folate Receptor Alpha (FRα)-Targeting Antibody-drug Conjugate (ADC) in Participants with Selected Tumor Types	Phase I/II	NCT04300556	Metastatic solid tumors, including NSCLC, that is refractory or not amenable to the standard of care therapy	Farletuzumab ecteribulin	FOLR1	Tubulin inhibitor	Treated and stable BM included; untreated BM or subdural disease excluded	Recruiting
	PRO1184 for Advanced Solid Tumors (PRO1184-001)	Phase I/II	NCT05579366	Locally advanced, unresectable or metastatic solid tumors, including NSCLC, that is refractory or not amenable to the standard of care therapy	PRO1184	FOLR1	Topoisomerase I inhibitor	Previously treated and stable BM included; active CNS disease excluded	Recruiting
	Safety of GQ1001 in Adult Patients with HER2-Positive Advanced Solid Tumors	Phase I	NCT04450732	Locally advanced or metastatic HER2-expressing solid tumors, including NSCLC, that is refractory or not amenable to the standard of care therapy	GQ1001	HER2	Pyrotinib, HER-TKI	Treated and asymptomatic BM included; untreated and symptomatic BM excluded	Recruiting
	DS8201a and Pembrolizumab in Participants with Locally Advanced/Metastatic Breast or Non-Small Cell Lung Cancer	Phase I	NCT04042701	Locally advanced or metastatic breast cancer or NSCLC with HER2-overexpression or *HER2*-mutant disease	Trastuzumab deruxtecan	HER2	Topoisomerase I inhibitor	Active CNS disease excluded	Recruiting
	A Study of Disitamab Vedotin in Previously Treated Solid Tumors That Express HER2	Phase II	NCT06003231	Locally advanced, unresectable or metastatic solid tumors, including NSCLC, that is refractory or not amenable to the standard of care therapy, has received prior PDL-(L)1, and has a HER2 overexpression	Disitamab vedotin	HER2	Auristatin microtubule inhibitor	Active and untreated CNS disease and LMD excluded	Recruiting
	A Study of SGN-B6A in Chinese Participants with Advanced Solid Tumors	Phase I	NCT06549816	Locally advanced, unresectable or metastatic solid tumors, including NSCLC, that is refractory or not amenable to the standard of care therapy	Sigvotatug vedotin	IB6	Auristatin microtubule inhibitor	Stable and treated BM included; active CNS disease excluded	Not yet recruiting
	A Study of SGN-MesoC2 in Advanced Solid Tumors	Phase I	NCT06466187	Locally advanced, unresectable or metastatic solid tumors, including NSCLC, that is refractory or not amenable to the standard of care therapy	SGN-MesoC2	MSLN	N/A	Stable and treated BM included; untreated BM and LMD excluded	Recruiting
	A Study of PHN-010 in Patients with Advanced Solid Tumors	Phase I	NCT06457997	Advanced or metastatic solid tumors, including NSCLC, that is refractory or not amenable to the standard of care therapy	PHN-010	NA	NA	Untreated CNS disease excluded	Recruiting
	A Study of LY4101174 in Participants with Recurrent, Advanced or Metastatic Solid Tumors	Phase I	NCT06238479	Locally advanced or metastatic solid tumors, including NSCLC, that is refractory or not amenable to the standard of care therapy	LY4101174	NECTIN4	Auristatin microtubule inhibitor	Known or suspected uncontrolled CNS disease excluded	Recruiting
	A Study of LY4052031 in Participants with Advanced or Metastatic Urothelial Cancer or Other Solid Tumors (NEXUS-01)	Phase I	NCT06465069	Locally advanced or metastatic solid tumors, including NSCLC, that is refractory or not amenable to the standard of care therapy	LY4052031	NECTIN4	Topoisomerase I inhibitor	Known or suspected uncontrolled CNS disease excluded	Recruiting
	Study BT8009-100 in Subjects with Nectin-4 Expressing Advanced Malignancies	Phase I/II	NCT04561362	Locally advanced, unresectable or metastatic solid tumors, including NSCLC, that is refractory or not amenable to the standard of care therapy	BT8009	NECTIN4	Auristatin microtubule inhibitor	Active and untreated CNS and LMD excluded	Recruiting
	CAB-ROR2-ADC Safety and Efficacy Study in Patients with TNBC or Head & Neck Cancer (Ph1) and NSCLC or Melanoma (Ph2)	Phase I/II	NCT03504488	Locally advanced, unresectable or metastatic solid tumors, including NSCLC, that is refractory or not amenable to the standard of care therapy	Ozuriftamab vedotin	ROR2	Auristatin microtubule inhibitor	Uncontrolled CNS disease excluded	Recruiting
	Study of XB002 in Subjects with Solid Tumors (JEWEL-101)	Phase I	NCT04925284	Locally advanced, unresectable, or metastatic solid tumors, including metastatic NSCLC, that is refractory or not amenable to the standard of care therapy	XB002	TF	Auristatin microtubule inhibitor	Treated BM included; untreated and active BM or cranial epidural disease excluded	Active, not recruiting
	A Study to Evaluate TROP2 ADC LCB84 Single Agent and in Combination with an Anti-PD-1 Ab in Advanced Solid Tumors	Phase I/II	NCT05941507	Advanced solid tumors, including NSCLC, that is refractory or not amenable to the standard of care therapy	LCB84	TROP2	Auristatin microtubule inhibitor	Stable and treated BM included; active and progressing CNS disease or LMD excluded	Recruiting
	A Phase 1/2 Study of OBI-992 in Subjects with Advanced Solid Tumors	Phase I/II	NCT06480240	Advanced or metastatic solid tumors, including NSCLC, that is refractory or not amenable to the standard of care therapy	OBI-992	TROP2	Topoisomerase I inhibitor	Treated and stable BM included; untreated CNS disease excluded	Recruiting
	Phase I-II, FIH, TROP2 ADC, Advanced Unresectable/Metastatic Solid Tumors, Refractory to Standard Therapies (A264)	Phase I/II	NCT04152499	Locally advanced or metastatic solid tumors, including NSCLC, that is refractory or not amenable to the standard of care therapy	Sacituzumab tirumotecan	TROP2	Topoisomerase I inhibitor	Symptomatic and active BM, history of LMD, brainstem metastasis, and spinal cord metastasis excluded	Recruiting

Abbreviations: ADC, antibody–drug conjugate; ALK, anaplastic lymphoma kinase; AXL, tyrosine-protein kinase receptor UFO; BM, brain metastases; CDH3, cadherin-3; CEACAM5, carcinoembryonic antigen-related cell adhesion molecule 5; CNS, central nervous system; EGFR, epidermal growth factor receptor; FOLR1, folate receptor alpha-1; HER2/3, human epidermal growth factor receptor 2/3; LMD, leptomeningeal disease; MET, hepatocyte growth factor; MSLN, mesothelin; NA, not available; NaPi2b, sodium-dependent phosphate transport protein 2B; NECTIN4, nectin cell adhesion molecule 4; NSCLC, non-small cell lung cancer; IB6, integrin beta-6; PD-(L)1, programmed cell death (ligand)1; PTK7, tyrosine-protein kinase-like 7; ROR2, receptor tyrosine kinase like orphan receptor 2; ROS1, ROS proto-oncogene 1; TF, human tissue factor; TKI, tyrosine kinase inhibitor; TROP2, trophoblast cell-surface antigen 2.
